# Pan-Domain Analysis of ZIP Zinc Transporters

**DOI:** 10.3390/ijms18122631

**Published:** 2017-12-06

**Authors:** Laura E. Lehtovirta-Morley, Mohammad Alsarraf, Duncan Wilson

**Affiliations:** 1 Aberdeen Fungal Group, Medical Research Council Centre for Medical Mycology, Institute of Medical Sciences, University of Aberdeen, Aberdeen AB25 2ZD, UK; L.Lehtovirta-Morley@uea.ac.uk (L.E.L.-M.); r01mjaa@abdn.ac.uk (M.A.); 2 School of Biological Sciences, University of East Anglia, Norwich NR4 7TJ, UK

**Keywords:** ZIP, zinc, transport, transporter, evolution, pathogenic fungi, *Candida albicans*

## Abstract

The ZIP (Zrt/Irt-like protein) family of zinc transporters is found in all three domains of life. However, little is known about the phylogenetic relationship amongst ZIP transporters, their distribution, or their origin. Here we employed phylogenetic analysis to explore the evolution of ZIP transporters, with a focus on the major human fungal pathogen, *Candida albicans*. Pan-domain analysis of bacterial, archaeal, fungal, and human proteins revealed a complex relationship amongst the ZIP family members. Here we report (i) a eukaryote-wide group of cellular zinc importers, (ii) a fungal-specific group of zinc importers having genetic association with the fungal zincophore, and, (iii) a pan-kingdom supercluster made up of two distinct subgroups with orthologues in bacterial, archaeal, and eukaryotic phyla.

## 1. Introduction

Zinc is an essential micronutrient for all living organisms. This is because many proteins (particularly enzymes and transcription factors) require zinc to function. In fact, 9% of eukaryotic proteins are predicted to interact with this metal. As well as acting as an essential cofactor for proteins involved in a large number of cellular processes, zinc can also be toxic if present in excess. Therefore, zinc acquisition, homeostasis, and detoxification is crucial for cell survival and proliferation. However, pathogenic microbes have to deal with extremes in zinc bioavailability due to the action of nutritional immunity. This term describes a variety of host processes which manipulate microbial exposure to trace metals, particularly zinc, iron, manganese, and copper. For example, following phagocytosis by macrophages, bacterial cells can face potential zinc and copper toxicity [1]. However, most examples of nutritional immunity involve host-driven metal sequestration together with microbial starvation [2].

Many bacterial pathogens utilise an ABC (ATP-binding cassette) transporter (ZnuABC) for high-affinity zinc uptake during infection. These systems consist of a substrate-binding protein ZnuA, permease ZnuB, and ATPase (ZnuC). An increasing body of literature is demonstrating an important role for ZnuABC-mediated zinc assimilation in bacterial pathogenicity. For a recent review on bacterial zinc assimilation, readers are directed to Capdevila et al. [3]. 

In contrast, with the exception of some recent studies in fungi, far less is known about how zinc homeostasis in eukaryotic pathogens influences their virulence. Zinc import via a ZnuABC-like system has not been reported in eukaryotes. Rather, they appear to predominantly employ ZIP-type transporters for cellular zinc import [4]. The name derives from fungal Zrt (zinc regulated transporter) [5] and plant Irt (iron-regulated transporter) [6] proteins.

However, the understanding of this ZIP-mediated zinc transport is complicated by the architecture of the eukaryotic cell. Unlike most bacteria and archaea, where cellular import only occurs at the plasma membrane, eukaryotic ZIPs can also deliver zinc from various intracellular organelles into the cytoplasm. Metal promiscuity may also confound phylogenetic interpretation: certain bacterial ZIPs have been shown to transport multiple metals. For example, *Salmonella enterica* ZupT transports both zinc and manganese [7]. In eukaryotes, different ZIP transporters can transport different metals. For example, *Saccharomyces cerevisiae* Atx2 is implicated in Golgi manganese homeostasis [8]. However, most eukaryotic ZIPs are implicated in zinc transport [4]. 

*S. cerevisiae* is one of the best understood models of eukaryotic ZIP-mediated zinc transport. This yeast encodes five ZIP transporters: two plasma membrane importers and three intracellular organellar transporters. There is an emerging and important role for ZIP transporters in the pathogenicity of human fungal pathogens [9,10,11,12], but little is known about the phylogeny, distribution, or origin of these transporters either within fungal pathogens or throughout different domains of life. Here we take advantage of OrthoMCL, to investigate the evolution of ZIP-type zinc transporters, with a focus on the medically important fungus *Candida albicans*. 

## 2. Phylogenetic Analysis of ZIP Transporter

In order to investigate their phylogenetic relationships, ZIP orthologue groups were constructed using OrthoMCL (Available online: http://orthomcl.org/orthomcl/) [13]. OrthoMCL generates orthologue superclusters from 36 Bacteria (including six Firmicutes and 19 Proteobacteria), 16 Archaea, 9 Euglenozoa (Trypanosomes, *Leishmania*), 4 Amoebae, 11 Viridiplantae (plants, algae), 15 Alveolates (e.g., Apicomplexa such as *Plasmodium*), 24 Fungi (including 4 Basidiomycetes, 3 Microsporidia, and 17 Ascomycetes), 29 Metazoa, and 6 miscellaneous Eukaryotes (Oomycete, *Giardia*) genomes.

This approach resulted in 38 orthologue groups with ZIP zinc transporter Pfam annotations. The majority of these contained only few poorly connected ZIP proteins, and several represented likely expansions in metazoans or Viridiplantae; that is, likely associated with the development of multicellularity. However, several superclusters of interest were identified, which we discuss below. 

## 3. Conserved Plasma Membrane Importer Cluster (OG5_126707)

**Figure 1:**
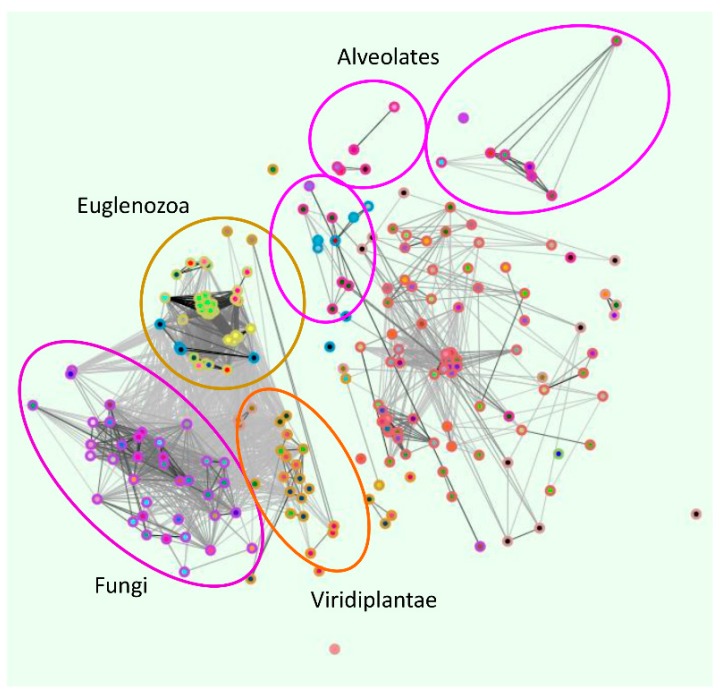
Conserved plasma membrane importer Cluster (OG5_126707). Clustering performed using OrthoMCL. Orthologues of *C. albicans* Zrt2 are conserved within eukaryotes. All characterised members of the clusters are implicated in plasma membrane zinc import.

OG5_126707 member ZIP transporters were exclusively eukaryotic and contained transporters from all studied eukaryotic groups. Figure 1 shows the cluster graph of OG5_126707. With the exception of the three Microsporidian species, fungal orthologues clustered together, as did Viridiplantae, Euglenozoa parasites (e.g., Trypanosomes), and to a lesser extent, Alveolates. Amoebic orthologues (blue circles) distributed between parasite clusters. The large cluster to the central right of Figure 1 includes Metazoan ZIP transporters. Not only were all eukaryotic groups represented in this supercluster, but most analysed species were also present. For example, 23 of the 24 analysed fungal species were represented.

Several members of the OG5_126707 supercluster have already been characterised. These include human ZIP1 and ZIP3, *S. cerevisiae* Zrt1 and Zrt2, *S. pombe* Zrt1, *A. fumigatus* ZrfA and ZrfB, *C. neoformans* Zip1 and Zip2, *C. albicans* Zrt2, and *Leishmania infantum*. Notably, all 11 of these transporters are implicated in cellular zinc import at the plasma membrane [5,11,14,15,16,17,18,19].

## 4. The Fungal Zincophore Locus Cluster (OG5_141027)

**Figure 2:**
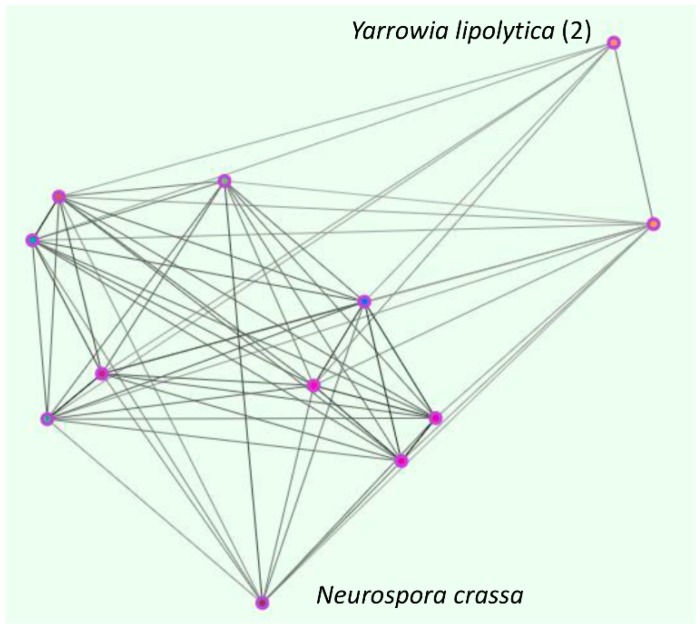
The fungal zincophore locus cluster (OG5_141027). Clustering performed using OrthoMCL. Orthologues of the zincophore-associated ZIP, Zrt1 (zinc regulated transporter 1) in *C. albicans* are specific to the fungal kingdom.

This cluster was unique to fungi (with the exception of two very loosely connected Trypanosomal sequences). However, unlike OG5_126707 which included 23 of the 24 fungal species, this cluster only contained 10 species. These included *C. albicans* (Zrt1) and *A. fumigatus* (ZrfC). Interestingly, both of these transporters have been reported to be up-regulated specifically at neutral/alkaline pH, and in the case of *C. albicans* Zrt1, to act as a cell surface docking protein for the secreted zincophore, Pra1 [20,21]. We have previously reported that the Zrt1 and Pra1 encoding genes are syntenic, not only in *C. albicans*, but in multiple fungal species [21,22]. Moreover, for *C. albicans* and *A. fumigatus*, the gene pairs are known to be co-expressed in response to zinc limitation [20,21]. Based on these observations, we have proposed that the *ZRT1*/*PRA1* locus may function as a conserved zincophore/receptor in multiple fungal species [22]. We therefore interrogated the genetic loci of those species identified in cluster OG5_141027. Gene order analysis revealed that seven of the 10 species here have maintained a syntenic relationship between orthologues of *C. albicans PRA1* and *ZRT1*. Two species—*Yarrowia lipolytica* and *Neurospora crassa*—have lost *PRA1*, and in one (*Gibberella zeae*, also called *Fusarium graminearum*), *PRA1*-*ZRT1* synteny has broken. One of the *PRA1*-negative species, *Yarrowia lipolytica*, has undergone duplication and divergence of the Zrt1 orthologue (Figure 2).

These observations are similar to our previous study—of 16 selected species analysed in Citiulo et al., 10 encoded *PRA1* orthologues and, of these 10 species, six maintained synteny with a *ZRT1* orthologue. To examine how widespread the syntenic relationship is, we interrogated the NCBI database. Of 102 species analysed, we identified Pra1 orthologues in 87 (85.3%) species and, of the Pra1^+^ species, 61 (70.1%) have maintained a syntenic relationship between *PRA1* and *ZRT1* (Table S1).

The fact that only ascomycete ZIPs were identified within this OrthoMCL cluster is probably due to the low number of basidiomycete species present in this database. In fact, BLASTp analysis of *C. albicans* Zrt1 against non-ascomycetes identified numerous ZIPs which reciprocally hit *C. albicans* Zrt1. Moreover, both ascomycete and basidiomycete species exhibit synteny of zincophore and ZIP orthologues (see [21] and Table S1).

While it should be pointed out that both *PRA1* [21] and *ZRT1* [22] orthologues have been lost multiple times throughout the fungal kingdom, this indicates that, when present, the genes tend to share a syntenic relationship. This most likely serves to simplify modular co-expression. The observations reported here support our earlier conclusion that *PRA1*-*ZRT1* synteny represents an ancient and highly successful adaption within the fungal kingdom [21].

## 5. The ZupT/ZIP11/Zrt3 Pan-Domain Supercluster (OG5_127397)

**Figure 3:**
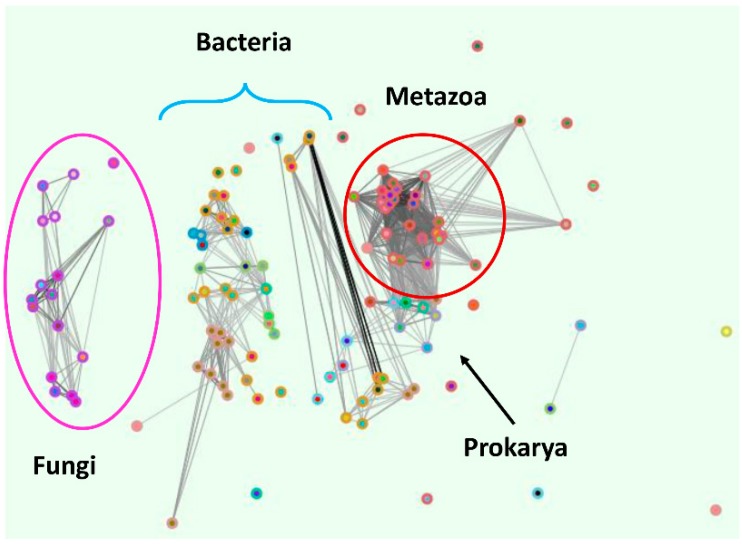
Fungal Zrt3, Prokaryote ZupT, Metazoan Zip11 pan-domain supercluster (OG5_127397). Clustering performed using OrthoMCL. Note the separation of eukaryotic (Fungi and Metazoan) subclusters by prokaryotic proteins.

The OG5_127397 supercluster (Figure 3) was the only cluster to contain ZIP proteins from all three domains of life. In fact, all phyla, with the exception of Alveolate and Euglenozoa parasites were represented.

A number of bacterial (16) and archaeal (5 or 6) members were present in the OG5_127397 supercluster. No archaeal ZIP transporters have been studied to-date. In bacteria, the Zip transporter, ZupT, has been characterised in *Escherichia coli*, *Cupriavidus metallidurans*, and *Salmonella enterica*. In all three species, a role in zinc import has been described [7,23,24]. *E. coli* ZupT appears to transport several other cations in addition to zinc [25], and *S. enterica* ZupT imports both zinc and manganese [7].

Orthologues were present throughout the fungal kingdom, but are absent from the Microsporidia and Basidiomycota. The *S. cerevisiae* member, Zrt3, has been shown to transport zinc out of the fungal vacuole [26], and our own work indicates that the *C. albicans* orthologue plays a similar role [27]. The human member, ZIP11, has been implicated in Golgi zinc transport [28]. 

Based on similarity between human ZIP11 and bacterial ZIP (ZupT) proteins, Yu et al. [27] have proposed that this family represents the most ancient ZIP [27], present in the last universal common ancestor. 

Similarly, the identification of *S. cerevisiae* Zrt3 led to the recognition of prokaryotic ZIP-type transporters in the first place, as Zrt3 (but not the previously characterised yeast Zrt1 and Zrt2) shared sequence similarity with bacterial and archaeal proteins [26]. 

In this context, the position of metazoan ZIP11, fungal Zrt3, and prokaryotic ZupT within the same supercluster is in line with an ancient origin [26,29]. 

Surprisingly, however, Fungal (Zrt3) and Metazoan (ZIP11) clusters were very distinct, and both had higher similarity to prokaryotic ZIPs than to each other (Figure 3). Furthermore, direct alignments showed that fungal Zrt3 and human ZIP11 shared only 28% sequence identity (e-value 3.1 × 10^−2^). This was unexpected, as within the Eukarya, fungi and metazoans are very closely related [30]. This suggests that fungal Zrt3 and metazoan ZIP11 may not be closely related. 

We therefore compared fungal (Zrt3-type) and metazoan (ZIP11-type) with more bacterial and archaeal sequences.

In order to capture bacterial and archaeal diversity as broadly as possible, fungal (*C. albicans* Zrt3) and metazoan (human ZIP11) sequences were subjected to individual BLASTp searches against Firmicutes, Proteobacteria, Actinobacteria, Spirochetes, Euryarchaeota, and Crenarchaeota species available at NCBI. These analyses identified predicted ZIP transporters in all six prokaryotic phyla. 

Sequence similarities between fungal Zrt3 and prokaryotic best hits were 30–40% (e-value 10^16^–10^15^) for bacteria, and even lower, ≤30% identity (e-value ~ 10^−12^), for archaea. Sequence similarity between fungal Zrt3 and bacterial proteins was limited to the C-terminal 200 amino acids. Indeed, when we repeated BLASTp analysis with the C-terminal 200 amino acids alone, we identified greater similarity (e-value 6 × 10^−19^). No sequence identity was observed for the N-terminal 391 amino acids out with the fungal kingdom.

We observed a higher degree of sequence similarity between Hs ZIP11 and bacterial and archaeal species (~45% identity, e-value 10^−81^–10^−60^).

**Figure 4:**
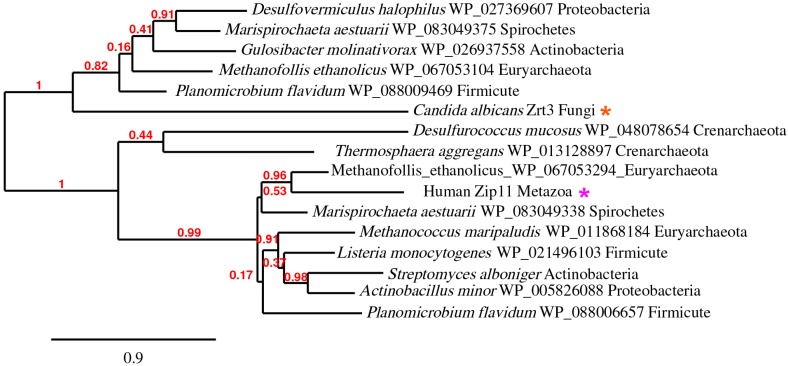
Pan-domain supercluster phylogeny. Phylogenetic tree generated using Phylogeny.fr (Available online: http://www.phylogeny.fr/). Note the presence of two branches with prokaryotic ZIPs related to both Fungal Zrt3 and Metazoan Zip11.

Next, we aligned fungal, mammalian, bacterial, and archaeal species’ ZIP sequences using Phylo.fr [31,32]. The resulting tree formed two distinct branches: one containing the fungal Zrt3 and the other, metazoan ZIP11 (Figure 4). Remarkably, both branches contained ZIP transporters from Firmicutes, Proteobacteria, Actinobacteria, Spirochete, and Euryarchaeota species, whilst the Human Zip11 branch rooted against the two identified Crenarchaeota. This suggests that prokaryotes have two different ZIP transporters: one related to fungal Zrt3 and the other to metazoan ZIP11.

If this is the case, we may anticipate the existence of extant prokaryotic species with both types. Indeed, the respective best hits of fungal Zrt3 and metazoan ZIP11 against Spirochetes were two independent ZIP transporters in the same species: *Marispirochaeta aestuarii* (Figure 4).

We therefore subjected human ZIP11 to BLASTp analysis against prokaryotic species which were identified in the fungal Zrt3 search, and vice versa. In most cases, we identified the same ZIP as in the previous search round, or the sequence similarity was too low to return a subject. However, when we queried fungal Zrt3 against the Firmicute *Planomicrobium flavidum* and the Euryarchaeota *Methanofollis ethanolicus* (two species which had metazoan ZIP11 orthologues), we identified independent ZIP transporters. The ZIP pairs from these three species clustered on the two distinct branches of the tree (Figure 4). Therefore, it would appear that these three prokaryotic species encode two independent ZIP transporters. 

This is interesting because it demonstrates the existence of two distinct classes of ZIP transporter in multiple prokaryotic phyla.

Although fungal Zrt3 and metazoan ZIP11 were identified as belonging to the same orthologue supercluster, their similarity was very low (identity 28%, e-value 3.1 × 10^−2^). Moreover, their relationship to distinct prokaryotic proteins (Figure 3 and Figure 4) is not suggestive of a close phylogenetic relationship. 

We therefore performed BLASTp analysis of fungal Zrt3 excluding the fungal kingdom (NCBI). Intriguingly, outside of the fungal kingdom, Zrt3 shares highest similarity with bacterial sequences and not with other Eukaryotes, as would be expected.

We therefore systematically analysed ZupT from *Desulfovermiculus halophilus* (which was one of the bacterial ZIPs with highest similarity to fungal Zrt3) against the major eukaryotic phyla. 

*D. halophilus* ZupT did not share sequence identity with any proteins within the Parabasalia, Diplomonadida, Ciliophora, or Euglenozoa. Only one species within the Heterolobosea (the Apicomplexa), and a handful of *Dictyostelium* and *Acytostelium* species within the Mycetozoa had proteins with similarity to *D. halophilus* ZupT (not shown). 

We retrieved a large number of hits from within the Heterokonta (e-value 7 × 10^−60^ [47% identity] to e-value 4 × 10^−26^ [28% identity]) and Viridiplantae (10^−50^, 43% identity) and, of those top hits analysed, they aligned to the fungal Zrt3 branch of Figure 4. Within the Metazoa, we did identify ZIP transporters with sequence similarity to *D. halophilus* ZupT, but (with the exception of *Oikopleura dioica*) these aligned to the human ZIP11 branch of the tree (not shown). 

Therefore, it appears that the origin of fungal Zrt3 is complex. It is possible that the gene was inherited vertically into the fungi, and that it has been lost multiple times within extant eukaryotic lineages. However, given the absence of Zrt3 orthologues from basal eukaryotes, its acquisition via horizontal gene transfer may represent an alternative explanation. 

We note that the observed similarities of ZIP11 and Zrt3 with prokaryotic proteins are in agreement with the conclusions of both MacDiarmid (2000) and Yu (2013) [26,29], that these proteins may represent ancient ZIP transporters in metazoans and in fungi, respectively. However, the diversity of bacterial and archaeal protein sequences within this orthologue supercluster (Figure 4) suggests that they arose from distinct genes. 

In summary, our analysis of fungal ZIP transporters indicates that there are three major orthologue groups with different degrees of conservation within and outside of the eukaryotes.

(i) A conserved group of eukaryotic proteins (OG5_126707) encompassing fungal, metazoan, and parasite plasma membrane importers; (ii) A fungal-specific group of zinc importers (OG5_141027), genetically associated with the fungal zincophore; (iii) A pan-domain supercluster (OG5_127397), formed of two distinct groups with orthologues in all three domains of life. 

At this stage, it is unclear whether eukaryotic members of this supercluster were inherited vertically or horizontally. However, our analyses indicate the presence of two relatively distinct groups of ZIP transporters in extant bacterial and archaeal species. Interestingly, the fungal members of this group appear to be involved in organellar (vacuolar) zinc export, rather than plasma membrane import.

Since the emergence of the Eukarya, ZIP transporter genes have clearly undergone multiple rounds of expansion. This is presumably to meet the requirements of an organellar lifestyle and, in the case of metazoans (humans for example have 14 ZIP family members), multicellularity.

Because zinc can be highly limited during infection due to the action of nutritional immunity, understanding the nature of pathogen (and host) zinc transporters may help inform future therapeutic or diagnostic strategies.

## Supplementary Materials

Supplementary materials can be found at www.mdpi.com/1422-0067/18/12/2631/s1. Table S1. ZIP and zincophore gene synteny. Table lists the presence (denoted by respective accession numbers) or absence (N) of *PRA1* and *ZRT1* orthologues in 102 fungal species (NCBI). In species where both genes are present, column 4 indicates syntenic (Y), or non-syntenic (N) relationship.
