# Supplementary material for: Pan-Domain Analysis of ZIP Zinc Transporters
Source: Int J Mol Sci. 2017 Dec 6;18(12):2631. doi: 10.3390/ijms18122631 (PMC5751234; doi:10.3390/ijms18122631)
Supplement: Supplementary file 1 [file ijms-18-02631-s001.pdf]

No	Species	*PRA1* orthologue [accession]	*PRA1*-ZIP synteny	ZIP Zrt1 best hit [accession]	
1	*Candida albicans SC5314*	XP_715420	y	XP_715421	
2	*C. dubliniensis*	XP_002420285	y	XP_002420284	
3	*C. maltosa*	EMG45831	y	EMG45830	
4	*C. orthopsilosis*	N	-	XP_003867188	
5	*C. parapsilosis*	N	-	CCE43791	
6	*C. tenuis*	N	-	XP_006687343	
7	*C. tropicalis*	XP_002549456	y	XP_002549455	
8	*C. auris*	XP_018167856	N	XP_018167047	
9	*C. intermedia*	SGZ52912	N	SGZ48790	
10	*C. boidinii*	OWB68967	N	OWB64932	
11	*C. tanzawaensis NRRL-17324*	N	-	XP_020067499	
12	*C. glabrata*	N	-	XP_449534	
13	*C. arabinofermentans NRRL-2248*	N	-	ODV82974	
14	*Millerozyma farinosa CBS 7064*	CCE80559	y	CCE80560	
15	*Debaryomyces fabryi*	XP_015465996	y	XP_015465995	
16	*Debaryomyces_hansenii_CBS767*	XP_457655	y	XP_457654	
17	*Hyphopichia burtonii NRRL Y-1933*	XP_020077368	y	XP_020077367	
18	*Scheffersomyces_stipitis_CBS_6054*	XP_001386401	y	XP_001386213	
19	*Meyerozyma guilliermondii ATCC 6260*	XP_001482986	*	XP_001483613	
20	*Lodderomyces elongisporus NRRL YB-4239*	N	-	XP_001523151	
21	*Spathaspora passalidarum NRRL Y-27907*	N	-	XP_007373231	
22	*Babjeviella inositovora NRRL Y-12698*	N	-	XP_018982225	
23	*Saccharomyces cerevisiae YJM1190*	AJT88464	N	AJV47561	
24	*Saccharomyces kudriavzevii IFO 1802*	EJT41755	*	n	
25	*Saccharomyces eubayanus*	XP_018219198	N	XP_018222000	
26	*Saccharomyces arboricola H-6*	EJS41803	*	EJS43846	
27	*Torulaspora delbrueckii*	XP_003681486	N	XP_003681450	
28	*Lachancea thermotolerans CBS 6340*	XP_002552739	N	XP_002554598	
29	*Kluyveromyces lactis*	XP_452206	N	XP_453785	
30	*Wickerhamomyces anomalus NRRL Y-366-8*	XP_019037544	Y	XP_019037545	
31	*Nadsonia fulvescens var. elongata DSM 6958*	ODQ66887	Y	ODQ66888	
32	*Cyberlindnera fabianii*	CDR44326	Y	CDR44330	
33	*Wickerhamomyces ciferrii*	XP_011277151	Y	XP_011277152	
34	*Cyberlindnera jadinii NRRL Y-1542*	XP_020069857	y	XP_019037545	
35	*Cryptococcus amylolentus CBS 6039*	XP_018989879	y	XP_018989878	

36	*Cryptococcus depauperatus CBS 7841*	ODN87535	y	ODN87536	
37	*Kwoniella dejecticola CBS 10117*	XP_018261282	y	XP_018261281	
38	*Kwoniella pini CBS 10737*	XP_019011269	y	XP_019011270	
39	*Kwoniella mangroviensis CBS 10435*	OCF57484	Y	OCF57485	
40	*Tsuchiyaea wingfieldii CBS 7118*	N	-	XP_019030547	
41	*Cryptococcus neoformans*	N	-	OWZ52593	
42	*Cryptococcus gattii CA1280*	N	-	KIR49314	
43	*Aspergillus nidulans FGSC A4*	XP_659436	y	XP_659436	
44	*Aspergillus bombycis*	XP_022386508	Y	XP_022386509	
45	*Aspergillus lentulus*	GAQ08121	y	GAQ08120	
46	*Aspergillus fumigatus*	XP_022399211	y	XP_751869	
47	*Aspergillus turcosus*	OXN35595	y	OXN35592	
48	*Aspergillus fischeri NRRL 181*	XP_001267052	y	XP_001267053	
49	*Aspergillus parasiticus SU-1*	KJK62992	Y	KJK63050	
50	*Aspergillus udagawae*	GAO83587	N	GAO90807	
51	*Aspergillus oryzae RIB40*	XP_001817942	y	XP_001817943	
52	*Aspergillus wentii DTO 134E9*	OJJ40798	y	OJJ40799	
53	*Aspergillus sydowii CBS 593.65*	OJJ64235	N	OJJ64233	
54	*Aspergillus flavus NRRL3357*	XP_002373106	y	XP_002373107	
55	*Aspergillus nomius NRRL 13137*	XP_015408011	y	XP_015408010	
56	*Aspergillus thermomutatus*	OXS12286	y	OXS12287	
57	*Aspergillus versicolor CBS 583.65*	OJI98327	N	OJI98325	
58	*Aspergillus glaucus CBS 516.65*	XP_022399211	N	XP_022405657	
59	*Aspergillus cristatus*	ODM16441	*	ODM23093	
60	*Aspergillus calidoustus*	CEL05170	y	CEL05169	
61	*Aspergillus clavatus NRRL 1*	XP_001271840	y	XP_001271839	
62	*Penicillium camemberti*	CRL17198	y	CRL17197	
63	*Penicillium solitum*	OQE01136	y	OQE01072	
64	*Penicillium polonicum*	OQD66641	y	OQD66428	
65	*Penicillium nordicum*	KOS41019	N	KOS41024	
66	*Penicillium expansum*	XP_016597257	y	XP_016597256	
67	*Penicillium vulpinum*	OQE07605	y	OQE07538	
68	*Penicillium italicum*	KGO71854	y	KGO71853	
69	*Penicillium coprophilum*	OQE46171	y	OQE46829	
70	*Penicillium griseofulvum*	KXG46698	y	KXG46699	
71	*Penicillium rubens Wisconsin 54-1255*	XP_002562292	y	XP_002562293	
72	*Penicillium flavigenum*	OQE30241	y	OQE30484	
73	*Penicillium brasilianum*	CEJ55628	y	CEJ55627	
74	*Penicillium antarcticum*	OQD81362	y	OQD81283	
75	*Penicillium oxalicum 114-2*	EPS27156	*	EPS30514	
76	*Penicillium nalgiovense*	OQE90320	y	OQE90300	
77	*Penicillium arizonense*	XP_022493837	y	XP_022494017	
78	*Penicillium freii*	KUM61209	y	KUM61210	
79	*Penicillium roqueforti FM164*	CDM33564	y	CDM33563	
80	*Penicillium digitatum Pd1*	XP_014531321	y	XP_014531321	
81	*Penicillium subrubescens*	OKP15198	y	OKP15199	
82	*Penicillium occitanis*	PCG97133	y	PCG97134	

83	*Spizellomyces punctatus DAOM BR117*	XP_016604344	N	XP_016609628	
84	*Histoplasma capsulatum H88*	N	-	EGC45219	
85	*Pneumocystis jirovecii RU7*	N	-	XP_018229860	
86	*Coccidioides posadasii C735 delta SOWgp*	XP_003070371	Y	XP_003070372	
87	*Coccidioides immitis RS*	XP_001241166	y	XP_001241167	
88	*Paracoccidioides brasiliensis*	N	-	ODH38939	
89	*Stachybotrys chlorohalonata IBT 40285*	KFA60783	N	KFA66915	
90	*Blastomyces dermatitidis ER-3*	EEQ91485	N	EEQ91531	
91	*Anthracocystis flocculosa PF-1*	XP_007882094	N	XP_007879588	
92	*Hypsizygus marmoreus*	KYQ40894	y	KYQ40893	
93	*Tilletia indica*	OAJ01445	y	A4X13_g5920	
94	*Kockovaella imperatae*	XP_021870272	y	BD324DRAFT_581562	
95	*Tilletiaria anomala UBC 951*	XP_013239746	-	no hit	
96	*Ustilago maydis 521*	XP_011389526	y	UMAG_03110	
97	*Leucosporidium creatinivorum*	ORY67941	N	ORY91603	
98	*Moesziomyces aphidis DSM 70725*	ETS61969	y	ETS61968	
99	*Pseudozyma hubeiensis SY62*	XP_012190163	y	XP_012190162	
100	*Rhodotorula graminis WP1*	XP_018271388	N	XP_018270067	
101	*Sporisorium scitamineum*	CDW96755	*	CDU26164	
102	*Kalmanozyma brasiliensis GHG001*	XP_016290366	y	XP_016290367	

*Syntenic relationship not clear
